# 2,2′,4,4′-Tetra­methyl-7,7′-diazenediylbis(1,8-naphthyridin-1-ium) bis­(perchlorate)

**DOI:** 10.1107/S1600536809019485

**Published:** 2009-06-06

**Authors:** Juan Mo, Li Yuan, Jian-Hua Liu, Wen Chen, Xin-Sheng Li

**Affiliations:** a College of Animal Husbandry and Veterinary Studies, Henan Agricultural University, Zhengzhou, Henan Province 450002, People’s Republic of China

## Abstract

In the title salt, C_20_H_20_N_6_^2+^·2ClO_4_^−^, the cation is disposed about a center of symmetry at the mid-point of the N=N bond. The 1,8-naphthyridine systems are planar and the ten atoms have a mean deviation of 0.01 Å from the least-squares plane. The two planar 1,8-naphthyridine units are parallel but extend in opposite directions from the diazene bridge. The 1,8-naphthyridine aminium groups inter­act with perchlorate O atoms through N—H⋯O hydrogen bonds.

## Related literature

For 1,8-naphthyridine and its derivatives, see: Baker & Norman (2004 ▶); Ferrarini *et al.* (1997 ▶); Gavrilova & Bosnich (2004 ▶); Stadie *et al.* (2007 ▶).
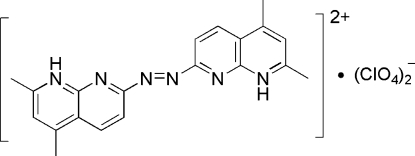


## Experimental

### 

#### Crystal data

C_20_H_20_N_6_^2+^·2ClO_4_^−^
*M* *_r_* = 543.32
Monoclinic, 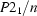

*a* = 8.2008 (16) Å
*b* = 13.042 (3) Å
*c* = 11.133 (2) Å
β = 102.63 (3)°
*V* = 1161.9 (4) Å^3^
*Z* = 2
Mo *K*α radiation
μ = 0.34 mm^−1^
*T* = 113 K
0.16 × 0.12 × 0.06 mm

#### Data collection

Bruker SMART CCD area-detector diffractometer
Absorption correction: multi-scan (*SADABS*; Sheldrick, 2003 ▶) *T*_min_ = 0.946, *T*_max_ = 0.975
9220 measured reflections
2669 independent reflections
2244 reflections with *I* > 2σ(*I*)
*R*_int_ = 0.042

#### Refinement

*R*[*F* ^2^ > 2σ(*F* ^2^)] = 0.045
*wR*(*F* ^2^) = 0.123
*S* = 1.06
2669 reflections
169 parameters
H atoms treated by a mixture of independent and constrained refinement
Δρ_max_ = 0.50 e Å^−3^
Δρ_min_ = −0.44 e Å^−3^

### 

Data collection: *SMART* (Bruker, 2000 ▶); cell refinement: *SAINT* (Bruker, 2000 ▶); data reduction: *SAINT*; program(s) used to solve structure: *SHELXS97* (Sheldrick, 2008 ▶); program(s) used to refine structure: *SHELXL97* (Sheldrick, 2008 ▶); molecular graphics: *SHELXTL* (Sheldrick, 2008 ▶); software used to prepare material for publication: *SHELXTL*.

## Supplementary Material

Crystal structure: contains datablocks global, I. DOI: 10.1107/S1600536809019485/hg2501sup1.cif

Structure factors: contains datablocks I. DOI: 10.1107/S1600536809019485/hg2501Isup2.hkl

Additional supplementary materials:  crystallographic information; 3D view; checkCIF report

## Figures and Tables

**Table 1:** Hydrogen-bond geometry (Å, °)

*D*—H⋯*A*	*D*—H	H⋯*A*	*D*⋯*A*	*D*—H⋯*A*
N1—H1⋯O1^i^	0.891 (10)	2.080 (15)	2.907 (2)	154
N1—H1⋯O3^i^	0.891 (10)	2.56 (2)	3.277 (3)	138

Symmetry code: (i) 
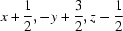
.

**Fig. 1.**
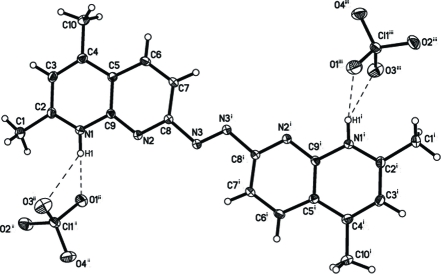
Molecular structure of the title compound showing the atom-numbering scheme and displacement ellipsoids drawn at the 40% probability level. The hydrogen bond is shown as a dashed line. [Symmetry codes: (i) 1 - x, 1 - y, 2 - z; (ii) 0.5 + x, 1.5 - y, -0.5 + z; (iii) 0.5 - x, -0.5 + y, 2.5 - z].

## supplementary crystallographic information

### Comment

1,8-Naphthyridine and its derivatives are used for binding of mismatched guanine or used as versatile ligands which are able to form metal aggregates with monodentates fashion or chelating bidentate fashion (Ferrarini *et al.*, 1997; Gavrilova & Bosnich, 2004; Baker & Norman, 2004; Stadie *et al.*, 2007). We report here a new 1,8-Naphthyridine compound, Fig. 1.

The title compound reveals 1,8-naphthyridine rings are linked by azo double bond, compound (I) is coplanar between two 1,8-naphthyridine rings. Each 1,8-naphthyridine ring is an almost planar group in which the ten atoms forming the 1,8-naphthyridine ring have mean deviation of 0.01 Å from the least-squares plane calculated using the ten atoms. The cation part sits on a center of symmetry at the mid-point of the N—N bond. To balance hydrogen ion charge of two 1,8-naphthyridine rings, there are two perchlorate groups in the crystal cell. The structure shows N-H···O hydrogen bonds between the amines ions of 1,8-naphthyridine groups and O atoms of perchlorate anions.

### Experimental

Single crystals of (I) suitable for an X-ray study were obtained by slow evaporation of an aqueous ethanol solution (30% *v*/*v*) in the presence of perchloric acid at 293 K over a period of four weeks.

### Refinement

Carbon bound hydrogen atoms were generated geometrically (C—H bond lengths of methylgroup fixed at 0.96Å, C—H bond lengths of naphthyridine fixed at 0.93Å), assigned appropriated isotropic thermal parameters, *U*_iso_(H) = 1.2*U*_eq_(C). The nitrogen proton was refined with the N—H  bond length of naphthyridine fixed at 0.89 Å and with *U*_iso_(H) = 1.5*U*_eq_(N).

### Crystal data

**Table:**

C_20_H_20_N_6_^2+^·2ClO_4_^−^	*F*(000) = 560
*M**_r_* = 543.32	*D*_x_ = 1.553 Mg m^−^^3^
Monoclinic, *P*2_1_/*n*	Mo *K*α radiation, λ = 0.71073 Å
Hall symbol: -P 2yn	Cell parameters from 3246 reflections
*a* = 8.2008 (16) Å	θ = 2.4–27.7°
*b* = 13.042 (3) Å	µ = 0.34 mm^−^^1^
*c* = 11.133 (2) Å	*T* = 113 K
β = 102.63 (3)°	Prism, yellow
*V* = 1161.9 (4) Å^3^	0.16 × 0.12 × 0.06 mm
*Z* = 2	

### Data collection

**Table:**

Bruker SMART CCD area-detector diffractometer	2669 independent reflections
Radiation source: fine-focus sealed tube	2244 reflections with *I* > 2σ(*I*)
graphite	*R*_int_ = 0.042
φ and ω scans	θ_max_ = 27.7°, θ_min_ = 2.4°
Absorption correction: multi-scan (*SADABS*; Sheldrick, 2003)	*h* = −8→10
*T*_min_ = 0.946, *T*_max_ = 0.975	*k* = −16→16
9220 measured reflections	*l* = −14→14

### Refinement

**Table:**

Refinement on *F*^2^	Primary atom site location: structure-invariant direct methods
Least-squares matrix: full	Secondary atom site location: difference Fourier map
*R*[*F*^2^ > 2σ(*F*^2^)] = 0.045	Hydrogen site location: inferred from neighbouring sites
*wR*(*F*^2^) = 0.123	H atoms treated by a mixture of independent and constrained refinement
*S* = 1.06	*w* = 1/[σ^2^(*F*_o_^2^) + (0.0581*P*)^2^ + 0.8149*P*] where *P* = (*F*_o_^2^ + 2*F*_c_^2^)/3
2669 reflections	(Δ/σ)_max_ = 0.001
169 parameters	Δρ_max_ = 0.50 e Å^−^^3^
0 restraints	Δρ_min_ = −0.44 e Å^−^^3^

### Special details

**Table:**

Geometry. All e.s.d.'s (except the e.s.d. in the dihedral angle between two l.s. planes) are estimated using the full covariance matrix. The cell e.s.d.'s are taken into account individually in the estimation of e.s.d.'s in distances, angles and torsion angles; correlations between e.s.d.'s in cell parameters are only used when they are defined by crystal symmetry. An approximate (isotropic) treatment of cell e.s.d.'s is used for estimating e.s.d.'s involving l.s. planes.
Refinement. Refinement of *F*^2^ against ALL reflections. The weighted *R*-factor *wR* and goodness of fit *S* are based on *F*^2^, conventional *R*-factors *R* are based on *F*, with *F* set to zero for negative *F*^2^. The threshold expression of *F*^2^ > σ(*F*^2^) is used only for calculating *R*-factors(gt) *etc*. and is not relevant to the choice of reflections for refinement. *R*-factors based on *F*^2^ are statistically about twice as large as those based on *F*, and *R*- factors based on ALL data will be even larger.

### Fractional atomic coordinates and isotropic or equivalent isotropic displacement parameters (Å^2^)

**Table:**

	*x*	*y*	*z*	*U*_iso_*/*U*_eq_	
N1	0.6259 (2)	0.83991 (12)	0.80976 (14)	0.0192 (4)	
N2	0.5576 (2)	0.68663 (12)	0.88762 (14)	0.0166 (3)	
N3	0.4967 (2)	0.52789 (12)	0.95394 (14)	0.0177 (3)	
C1	0.6950 (3)	0.99037 (16)	0.70209 (19)	0.0290 (5)	
H1A	0.7525	0.9466	0.6557	0.044*	
H1B	0.7573	1.0526	0.7228	0.044*	
H1C	0.5860	1.0063	0.6537	0.044*	
C2	0.6780 (3)	0.93708 (15)	0.81696 (17)	0.0198 (4)	
C3	0.7132 (3)	0.98554 (15)	0.93190 (18)	0.0214 (4)	
H3	0.7480	1.0536	0.9372	0.026*	
C4	0.6978 (2)	0.93524 (15)	1.03682 (17)	0.0199 (4)	
C5	0.6409 (2)	0.83139 (14)	1.02677 (16)	0.0167 (4)	
C6	0.6177 (3)	0.77045 (15)	1.12667 (17)	0.0196 (4)	
H6	0.6356	0.7978	1.2056	0.024*	
C7	0.5685 (3)	0.67085 (15)	1.10573 (16)	0.0191 (4)	
H7	0.5524	0.6293	1.1701	0.023*	
C8	0.5425 (2)	0.63213 (14)	0.98489 (15)	0.0156 (4)	
C9	0.6065 (2)	0.78426 (14)	0.91017 (16)	0.0156 (4)	
C10	0.7442 (3)	0.98621 (16)	1.15997 (19)	0.0291 (5)	
H10A	0.8410	0.9532	1.2088	0.044*	
H10B	0.6529	0.9809	1.2008	0.044*	
H10C	0.7686	1.0572	1.1493	0.044*	
Cl1	0.07021 (6)	0.72566 (4)	0.98120 (4)	0.02260 (17)	
O1	0.1777 (3)	0.76617 (13)	1.09150 (15)	0.0440 (5)	
O2	0.1613 (2)	0.65386 (14)	0.92339 (14)	0.0372 (4)	
O3	−0.0642 (2)	0.67208 (14)	1.01881 (17)	0.0407 (5)	
O4	0.0056 (2)	0.80751 (14)	0.89857 (15)	0.0380 (4)	
H1	0.607 (3)	0.8059 (18)	0.7387 (15)	0.037 (7)*	

### Atomic displacement parameters (Å^2^)

**Table:**

	*U*^11^	*U*^22^	*U*^33^	*U*^12^	*U*^13^	*U*^23^
N1	0.0273 (9)	0.0158 (8)	0.0153 (7)	−0.0011 (7)	0.0063 (6)	−0.0005 (6)
N2	0.0196 (8)	0.0150 (8)	0.0155 (7)	−0.0003 (6)	0.0049 (6)	0.0008 (6)
N3	0.0214 (8)	0.0162 (8)	0.0157 (7)	−0.0016 (6)	0.0043 (6)	0.0014 (6)
C1	0.0447 (14)	0.0198 (10)	0.0250 (10)	−0.0061 (9)	0.0129 (10)	0.0031 (8)
C2	0.0226 (10)	0.0160 (9)	0.0216 (9)	0.0005 (8)	0.0068 (7)	0.0031 (7)
C3	0.0240 (10)	0.0153 (9)	0.0248 (9)	−0.0026 (8)	0.0049 (8)	−0.0002 (7)
C4	0.0204 (10)	0.0182 (9)	0.0196 (9)	0.0002 (8)	0.0011 (7)	−0.0022 (7)
C5	0.0167 (9)	0.0162 (9)	0.0163 (8)	0.0016 (7)	0.0013 (7)	−0.0004 (7)
C6	0.0232 (10)	0.0221 (10)	0.0132 (8)	0.0010 (8)	0.0031 (7)	−0.0003 (7)
C7	0.0244 (10)	0.0198 (9)	0.0131 (8)	−0.0002 (8)	0.0043 (7)	0.0021 (7)
C8	0.0170 (9)	0.0154 (9)	0.0142 (8)	0.0001 (7)	0.0031 (7)	0.0009 (6)
C9	0.0169 (9)	0.0154 (9)	0.0154 (8)	0.0015 (7)	0.0052 (7)	0.0014 (7)
C10	0.0414 (13)	0.0221 (10)	0.0213 (10)	−0.0069 (10)	0.0011 (9)	−0.0048 (8)
Cl1	0.0238 (3)	0.0264 (3)	0.0174 (2)	0.00234 (19)	0.00408 (19)	−0.00095 (17)
O1	0.0653 (13)	0.0319 (9)	0.0272 (9)	−0.0120 (9)	−0.0067 (8)	−0.0026 (7)
O2	0.0414 (10)	0.0470 (10)	0.0264 (8)	0.0175 (8)	0.0142 (7)	−0.0006 (7)
O3	0.0293 (9)	0.0441 (11)	0.0529 (11)	0.0024 (8)	0.0183 (8)	0.0112 (8)
O4	0.0429 (11)	0.0393 (10)	0.0317 (8)	0.0102 (8)	0.0081 (8)	0.0129 (7)

### Geometric parameters (Å, °)

**Table:**

N1—C2	1.334 (3)	C4—C10	1.496 (3)
N1—C9	1.371 (2)	C5—C9	1.408 (2)
N1—H1	0.891 (10)	C5—C6	1.414 (3)
N2—C8	1.324 (2)	C6—C7	1.365 (3)
N2—C9	1.342 (2)	C6—H6	0.9300
N3—N3^i^	1.249 (3)	C7—C8	1.409 (2)
N3—C8	1.432 (2)	C7—H7	0.9300
C1—C2	1.489 (3)	C10—H10A	0.9600
C1—H1A	0.9600	C10—H10B	0.9600
C1—H1B	0.9600	C10—H10C	0.9600
C1—H1C	0.9600	Cl1—O4	1.4329 (17)
C2—C3	1.400 (3)	Cl1—O2	1.4348 (16)
C3—C4	1.370 (3)	Cl1—O3	1.4423 (17)
C3—H3	0.9300	Cl1—O1	1.4460 (17)
C4—C5	1.429 (3)		
			
C2—N1—C9	123.14 (16)	C7—C6—H6	120.4
C2—N1—H1	121.3 (18)	C5—C6—H6	120.4
C9—N1—H1	115.5 (18)	C6—C7—C8	118.79 (17)
C8—N2—C9	115.72 (15)	C6—C7—H7	120.6
N3^i^—N3—C8	113.16 (19)	C8—C7—H7	120.6
C2—C1—H1A	109.5	N2—C8—C7	124.55 (17)
C2—C1—H1B	109.5	N2—C8—N3	112.30 (15)
H1A—C1—H1B	109.5	C7—C8—N3	123.16 (16)
C2—C1—H1C	109.5	N2—C9—N1	115.66 (16)
H1A—C1—H1C	109.5	N2—C9—C5	125.33 (16)
H1B—C1—H1C	109.5	N1—C9—C5	119.01 (17)
N1—C2—C3	118.89 (17)	C4—C10—H10A	109.5
N1—C2—C1	118.73 (17)	C4—C10—H10B	109.5
C3—C2—C1	122.37 (18)	H10A—C10—H10B	109.5
C4—C3—C2	121.63 (18)	C4—C10—H10C	109.5
C4—C3—H3	119.2	H10A—C10—H10C	109.5
C2—C3—H3	119.2	H10B—C10—H10C	109.5
C3—C4—C5	118.47 (17)	O4—Cl1—O2	110.72 (10)
C3—C4—C10	121.09 (18)	O4—Cl1—O3	110.40 (11)
C5—C4—C10	120.42 (17)	O2—Cl1—O3	108.64 (11)
C9—C5—C6	116.46 (17)	O4—Cl1—O1	110.13 (11)
C9—C5—C4	118.84 (17)	O2—Cl1—O1	109.81 (12)
C6—C5—C4	124.70 (17)	O3—Cl1—O1	107.05 (12)
C7—C6—C5	119.10 (17)		
			
C9—N1—C2—C3	−0.7 (3)	C9—N2—C8—N3	−177.58 (16)
C9—N1—C2—C1	179.94 (19)	C6—C7—C8—N2	−2.1 (3)
N1—C2—C3—C4	1.0 (3)	C6—C7—C8—N3	177.69 (18)
C1—C2—C3—C4	−179.7 (2)	N3^i^—N3—C8—N2	171.1 (2)
C2—C3—C4—C5	−1.3 (3)	N3^i^—N3—C8—C7	−8.7 (3)
C2—C3—C4—C10	176.8 (2)	C8—N2—C9—N1	178.50 (17)
C3—C4—C5—C9	1.3 (3)	C8—N2—C9—C5	−0.4 (3)
C10—C4—C5—C9	−176.81 (19)	C2—N1—C9—N2	−178.19 (18)
C3—C4—C5—C6	−179.40 (19)	C2—N1—C9—C5	0.7 (3)
C10—C4—C5—C6	2.4 (3)	C6—C5—C9—N2	−1.6 (3)
C9—C5—C6—C7	1.7 (3)	C4—C5—C9—N2	177.76 (18)
C4—C5—C6—C7	−177.61 (19)	C6—C5—C9—N1	179.63 (17)
C5—C6—C7—C8	0.0 (3)	C4—C5—C9—N1	−1.1 (3)
C9—N2—C8—C7	2.2 (3)		

Symmetry codes: (i) −*x*+1, −*y*+1, −*z*+2.

### Hydrogen-bond geometry (Å, °)

**Table:**

*D*—H···*A*	*D*—H	H···*A*	*D*···*A*	*D*—H···*A*
N1—H1···O1^ii^	0.89 (1)	2.08 (2)	2.907 (2)	154
N1—H1···O3^ii^	0.89 (1)	2.56 (2)	3.277 (3)	138

Symmetry codes: (ii) *x*+1/2, −*y*+3/2, *z*−1/2.

## References

[bb1] Baker, R. S. & Norman, R. E. (2004). *Acta Cryst.* E**60**, m1761–m1763.

[bb2] Bruker (2000). *SMART* and *SAINT* Bruker AXS Inc., Madison, Wisconsin, USA.

[bb3] Ferrarini, P. L., Mori, C., Badawneh, M., Manera, C., Martinelli, A., Miceli, M., Ramagnoli, F. & Saccomanni, G. (1997). *J. Heterocycl. Chem.* **34**, 1501–1504.

[bb4] Gavrilova, E. L. & Bosnich, B. (2004). *Chem. Rev.* **104**, 349–383. 10.1021/cr020604g 14871128

[bb5] Sheldrick, G. M. (2003). *SADABS* Bruker AXS Inc., Madison, Wisconsin, USA.

[bb6] Sheldrick, G. M. (2008). *Acta Cryst.* A**64**, 112–122. 10.1107/S0108767307043930 18156677

[bb7] Stadie, N. P., Sanchez-Smith, R. & Groy, T. L. (2007). *Acta Cryst.* E**63**, m2153–m2154.

